# Supplementary material for: Physical Activity and Sedentary Behaviour Patterns in 326 Persons with COPD before Starting a Pulmonary Rehabilitation: A Cluster Analysis
Source: J Clin Med. 2019 Aug 29;8(9):1346. doi: 10.3390/jcm8091346 (PMC6780222; doi:10.3390/jcm8091346)
Supplement: Supplementary file 1 [file jcm-08-01346-s001.zip › Supplementary Table S1.docx]

**Supplementary Table S1.** The potential accelerometer indicators as a basis for the cluster identification.

| **Intensity Category** | **MET** | **Cut points** | **M (SD)** |
| --- | --- | --- | --- |
| Sedentary behaviour | < 1.5 | 0 - 99 | 559.38 (92.86) |
| Very light PA behaviour | 1.5 - 2.0 | 100 – 928 | 149.93 (50.20) |
| Light PA behavior | 2.0 - 3.0 | 929 – 1951 | 49.12 (24.76) |
| Moderate PA behaviour | 3.0 - 6.0 | 1952 – 5724 | 28.77 (22.33) |
| Vigorous PA behaviour* | 6.0 - 9.0 | 5725 – 9498 | 0.39 (1.60) |
| Very vigorous PA behaviour* | > 9.0 | > 9499 | 0.02 (0.22) |

Note: *not used as cluster formation variable
