# Supplementary figures and images for: Physical Activity and Sedentary Behaviour Patterns in 326 Persons with COPD before Starting a Pulmonary Rehabilitation: A Cluster Analysis
Source: J Clin Med. 2019 Aug 29;8(9):1346. doi: 10.3390/jcm8091346 (PMC6780222; doi:10.3390/jcm8091346)

**Supplementary Figure S2.** Scree plot for the identification of the number of clusters.


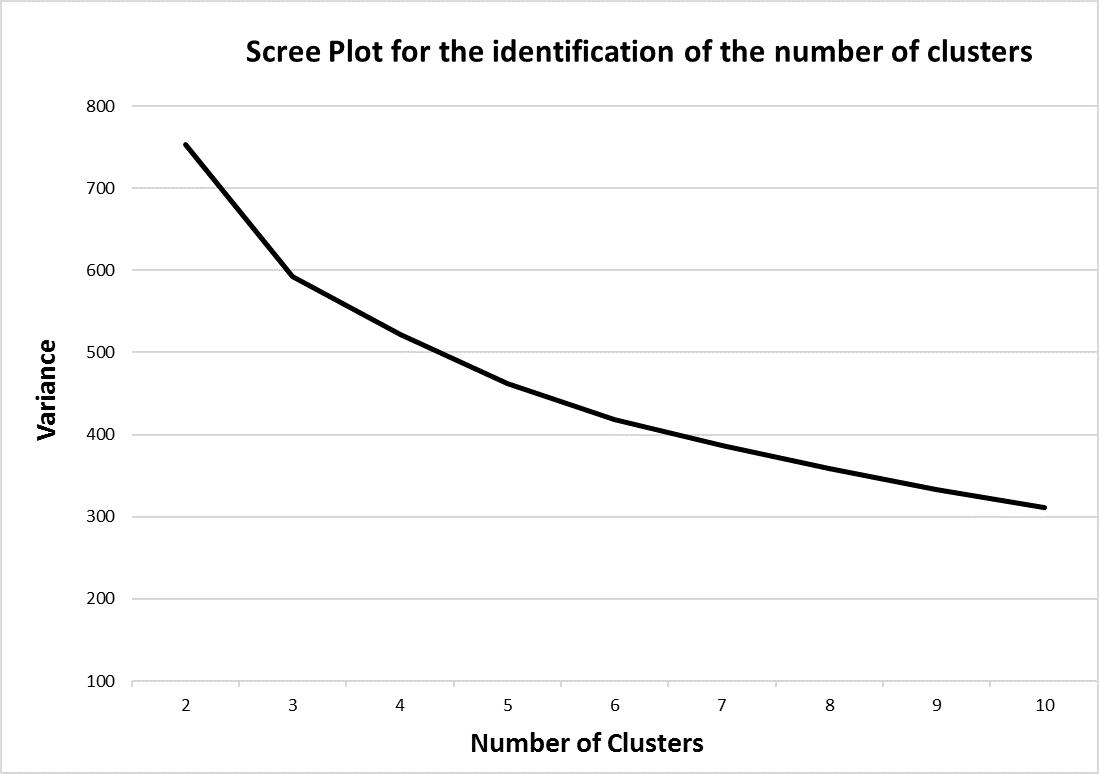

Supplement: Supplementary file 1 [file jcm-08-01346-s001.zip › Supplementary Figure S2.docx]
